# Depression, anxiety, and loneliness among adolescents and young adults with IBD in the UK: the role of disease severity, age of onset, and embarrassment of the condition

**DOI:** 10.1007/s11136-020-02653-9

**Published:** 2020-09-30

**Authors:** Pamela Qualter, Alison Rouncefield-Swales, Lucy Bray, Lucy Blake, Steven Allen, Chris Probert, Kay Crook, Bernie Carter

**Affiliations:** 1 Institute of Education, University of Manchester, Manchester Oxford Road, Manchester, M13 9PL UK; 2 Children, Young People and Families, Faculty of Health, Social Care & Medicine, Edge Hill University, St Helens Road, Ormskirk, L39 4QP UK; 3 Liverpool School of Tropical Medicine, Liverpool, UK; 4 Institute of Translational Medicine, University of Liverpool, Liverpool, UK; 5 St Marks & Northwick Park, London North West University Healthcare NHS Trust, London, UK

**Keywords:** IBD, Crohn’s, Ulcerative colitis, Mental health, Loneliness, Embarrassment

## Abstract

**Purpose:**

Adolescents and young adults (AYA) with Inflammatory Bowel Disease (IBD) report higher depressive symptoms and anxiety compared to healthy controls, with disease severity and abdominal pain being important factors. In the current study, building on what young people had told us in our previous work, we examined whether embarrassment of the condition, social self-efficacy, and friendship quality mediated the relationship between abdominal pain and disease severity, and mental health/well-being. We also included loneliness as a component of well-being.

**Methods:**

Data on depression, anxiety, loneliness, friendship quality, social self-efficacy, and disease embarrassment were collected from 130 AYA with IBD ages 14–25 years; data on disease severity and abdominal pain were taken from their medical records. Structural Equation Modeling (SEM) was used to test the relationships between the variables.

**Results:**

Using SEM, we established that higher IBD disease activity negatively impacted how AYA felt about their friendships and how embarrassed they were about their condition; embarrassment then influenced reports of mental health, including loneliness. Abdominal pain, disease onset, and social self-efficacy directly predicted internalising problems.

**Conclusion:**

In this sample of 14–25-year-old patients with IBD, specifics about the disease (severity and pain) predicted poorer mental health, suggesting discussion of mental health should be part of the clinical dialogue between patient and consultant. In addition, embarrassment about their condition increased depression, anxiety, and loneliness, mediating the relationship between disease severity and well-being. Thus, it is important to consider how perceived stigma affects those with chronic illness, and those issues should be explored in clinic.

## What is IBD

Inflammatory Bowel Disease (IBD) is a ‘chronic, heterogeneous, relapsing and remitting condition primarily as a consequence of inflammation within the bowel lumen’ [1]. IBD includes Crohn’s disease (CD) and ulcerative colitis (UC), and is a lifelong disease marked by episodes of remission and relapse [2]. IBD is characterised by uncertainty, unpredictability, and the intrusiveness of symptoms [3, 4]; symptoms most commonly include diarrhoea, abdominal pain, weight loss, blood in the stools, and fatigue [5]. The incidence of IBD in all age groups is increasing worldwide [2, 6], although the reason is unknown [7]. Worldwide, Europe has the highest reported prevalence values for IBD (UC, 505 per 100,000 persons; CD, 322 per 100,000 persons) and North America (UC, 249 per 100,000 persons; CD, 319 per 100,000 persons) [6]. The incidence is higher in the north compared to the south of Europe [8] and in western Europe compared to eastern Europe [9]. The incidence of paediatric-onset IBD is also rising [10].

## IBD and mental health

Individuals with IBD experience potential challenges to their psychological well-being: the course of the disease is unpredictable, the treatment and management regimes frustrating. Patients talk about the symptoms of the disease often being embarrassing and socially limiting [3, 11], which makes telling others about their condition difficult [12, 13]. While there are few studies with adolescents and young adults (AYA) with IBD, those studies show high rates of anxiety and depression among that population [14]. Only certain studies utilise a control group and those show that depression and anxiety are considerably higher among AYA with IBD than control groups of peers without chronic medical conditions [15]; they are comparable to other groups of AYA with chronic conditions, such as cystic fibrosis (CF), diabetes, and cancer [15], although rates of lifetime depression, but not anxiety, have been found to be higher among those with IBD compared to those with CF [16].

Most of the research on mental health among IBD patients is focused on depressive symptoms, with some studies also considering anxiety among AYA. However, missing from the discussion is whether AYA with IBD also experience loneliness. Loneliness is an important issue raised by adolescents with chronic diseases [17], and meta-analytic findings show that AYA with medical conditions are vulnerable to loneliness [18, 19]: AYA talk about feelings of disconnection from peers, that such disconnection impacts their quality of life, and is part of their poor mental health. To date, however, there has been no published work on loneliness among AYA with IBD. Loneliness in the general population of the AYA in the UK impacts employment prospects, with lonelier young people more likely to be out of work [20]; they also have difficulties coping with stress [20]. The current study is designed to fill the gap in our knowledge about loneliness among AYA with IBD. Given the associations between loneliness, depressive symptoms, and anxiety among AYA [21], an exploration of how IBD is associated with all three internalising problems is needed.

## Risk factors for poor mental health among AYA with IBD

Given that mental health problems impact quality of life and adherence to medication for those with IBD [22], it is important to understand risk factors for mental health problems for those with IBD. Findings from empirical studies suggest that, for IBD among AYA, disease severity (activity and pain) has an impact on mental health and well-being, although that effect is found in some studies, but not others [23]. In studies that found an effect, paediatric patients with IBD who reported greater disease activity and more pain also reported poorer mental health and well-being compared to peers with IBD in remission [24]: functional impairment and the aggressive treatment regimens for IBD contribute to negative affect.

Disease severity accounts for a small amount of variation in reports of well-being and mental health [25], suggesting that other factors are important. Given that those with IBD also report social problems more frequently than healthy peers [23], it is possible that problems with social relationships are key predictors of mental health problems among AYA with IBD. There is a social taboo related to bowel symptoms in most cultures, so IBD is susceptible to condition-related stigma [26], which means those with IBD report high levels of shame/embarrassment associated with having the condition [11, 27]. Fears of abdominal pain, bowel noises, faecal incontinence, and/or a bowel urgency in public mean many people with IBD withdraw from social interactions because they feel ashamed. IBD nondisclosure due to embarrassment is also a reported problem in the literature [13, 26], but, because concealment of any condition is associated with reduced engagement with others [28], feelings of disconnection and negative affect are likely to be high among those with IBD because of social problems as much as they are to do with disease severity. Indeed, recent work by Roberts, Gamwell et al. [29] showed that difficulty communicating about their illness to others was associated with thwarted belonging that was also linked to higher depression. Thus, where stigmatised beliefs are internalised to become part of one’s identity, there are potential impacts on mental health [28]: feeling ashamed of one’s self because of their IBD is likely to increase feelings of disconnection and depression. Gambin and Sharp [30] point out that AYA are particularly vulnerable to self-consciousness, which impacts their mental health; given too, that heightened shame and low social self-efficacy during this time increase feelings of loneliness [19], it seems that AYA with IBD may be particularly at risk of internalising problems. Thus, in the current study we explore how feelings of embarrassment of IBD impact mental health, including loneliness, controlling for social self-efficacy and friendship quality.

## The current study

The current study explores [1] whether, among adolescents and young adults (AYA) with IBD, disease symptoms are associated with poor mental health and well-being, including loneliness, and [2] examines how embarrassment of the condition, social self-efficacy, and friendship quality influence loneliness and whether they mediate the relationship between abdominal pain and disease severity, and mental health.

## Method

### Participants and procedure

The study was a cross-sectional questionnaire design. AYA, ages 14 to 25 years, with Inflammatory Bowel Disease (IBD) were invited to participate. They were patients attending the outpatient clinics or day units in three hospitals (two in Liverpool and one in London, United Kingdom). Eligibility criteria consisted of being between the ages of 14 and 25 years, with a diagnosis of IBD and beyond the first 3 months after diagnosis, able to provide assent/consent to participate, and able to complete the questionnaires and converse in English. Patients were excluded if they were judged by a clinician as too unwell and/or distressed to participate. Those with new-onset IBD were not included in the study because we saw that as a period of adjustment and did not want to add extra burden. AYA for the study were identified and screened by a research nurse; if they expressed interest in the study, a trained researcher approached the young person (and parent, as appropriate) and explained the study and provided them with an appropriate participant information sheet (PIS). They were then given a minimum of 20 min to think about the information before the researcher returned to ask if they were still interested and wished to participate in the study. Informed consent/assent was then sought by that researcher and documented using the appropriate consent and assent forms. All young people were able to provide consent or, if deemed more appropriate, assent with their parent/guardian providing consent. All young people and their parents provided written consent/assent.

130 young people with IBD consented to take part in the study. This was a response rate of 78%. To ensure patient confidentiality, and for the purpose of database management, clinical data and completed questionnaires were numerically coded, providing anonymity. The research nurses collated clinical data including demographics, age of diagnosis, IBD classification, disease activity scores, and symptoms for each participant. Ethics approval for the study was obtained from the North West-Liverpool East Research Ethics Committee (18/NW/0178) and research ethics committees at Edge Hill University and the University of Manchester. Research approval was granted by all participating sites.

### Measures

AYA in the ‘Being Me with IBD’ study completed questionnaires that measured mental health as follows: loneliness, depressive symptoms, and anxiety. They also completed questionnaires that examined friendship quality, social self-efficacy, and how their embarrassment of the condition impacted their relationships. Questionnaires were completed on iPads or on paper, depending on the participant’s preference. The questionnaires took approximately 10–15 min to complete.

#### Demographic and clinical variables

Current age, gender, and age of onset of IBD symptoms were recorded. Research nurses also provided data on IBD classification and disease activity scores using Harvey-Bradshaw Index (HBI)—Simple Index of Crohn’s Disease Activity (for those age 17 and above with Crohn’s Disease), Simple Clinical Colitis Activity Index (SCCAI) (for those age 17 and above with ulcerative colitis or indeterminate colitis), Paediatric Ulcerative Colitis Activity Index (PUCAI) (for those age 16 and below with Ulcerative Colitis or IBD-unclassified) and Weighted Paediatric Crohn’s Disease Activity Index (wPCDAI) (for those age 16 and below with Crohn’s Disease). Using those scales enabled IBD activity to be compared across the different age groups using the classification Remission, Mild disease, Moderate disease, and severe disease. Research nurses also provided data on abdominal pain. The HBI scores abdominal pain on a 0–3 scale, PUCAI on a 0, 5, 10 scale, and the wPCDAI on a scale of 0, 10, 20. For analysis, all pain scores were reclassified as 0, 1, or 2 (no pain, mild pain, moderate/severe pain). Pain scores for all patients with ulcerative colitis/IBD-unclassified colitis were not collected through the SCCAI, so not all patients were included in those sections of our analyses. Patient characteristics are provided in Table 1.

**Table 1:** Demographics of current sample

Characteristics	Range/response scale	Mean (SD) or %
Age (years)	14.00–25.00	18.69 (3.65)
Mean age at onset of IBD	4.00–25.00	14.17 (4.15)
Males %		42%
White British		84%
Ulcerative Colitis		31%
Crohn’s disease		64%
IBD-unclassified		5%
Current abdominal pain: no pain		57%
Current abdominal pain: mild pain		18%
Current abdominal pain: moderate/severe pain		2%
Current disease activity: remission^†^		55%
Current disease activity: mild^†^		29%
Current disease activity: moderate/severe^†^		10%
Mean GAD-7 score	0.00–20.00	6.29 (5.31)
Mean PHQ-8 score	0.00–21.00	6.52 (5.42)
Mean UCLA score	2.28–9.00	3.83 (1.26)
Mean friendship function score	0.25–4.04	3.36 (0.76)
Mean social self-efficacy score	1.56–5.00	3.18 (0.77)
IMPACT III embarrassment scale score	2.33–4.67	3.91 (0.47)

^†^SCCAI simple clinical colitis activity index (SCCAI) was categorised as remission (SCCAI ≤ 2), mild disease (SCCAI 3–5), moderate disease (SCCAI 6–11) and severe disease (SCCAI ≥ 12); 6% of AYA did not have data for this variable. This made the disease activity indicators/groups comparable for Adults with Crohn’s (measured using HBI), children with colitis (measured using the PUCAI), and children with Crohn’s (measured using wPCDAI) because they all use the same scale of remission, mild disease, moderate disease, and severe disease activity

#### Mental health variables

Validated measures of anxiety, depressive symptoms, and loneliness were administered. **Anxiety** was measured using the GAD-7 [31, 32]. GAD-7 is a seven-item measure of anxiety symptoms. Each item is rated using a four-point response scale from 0 (not at all) to 3 (nearly every day), giving a severity score between 0 and 21. A score above 7 is recommended to identify a likely anxiety disorder. **Depression** was measured using the Patient Health Questionnaire 8 (PHQ-8), a measure of depressive symptoms. PHQ-8 is the same as the PHQ-9, but without the item ‘How often have you been bothered by thoughts that you would be better off dead or of hurting yourself in some way? and includes the same response options as for the GAD-7. The presence of Item 9, asking about suicidal ideation, has been highlighted as a potential problem [33]. However, Wu et al. [34] have shown its removal has minimal influence on depression score, but reduces the number of false positives from people who endorse this item but would not be considered to be at risk for suicide based on measures intended to assess suicide risk. Total severity scores for the PHQ-8 are between 0 and 27. A score greater than 9 indicates clinically significant depression. The PHQ-9 is well validated against standard criteria, demonstrates sensitivity to change, and is used in a variety of clinical settings [31, 35, 36]. PHQ-9 and GAD-7 form part of the UK Department of Health’s National Minimum Data Set [37]; the National Institute for Health and Care Excellence supports their use for assessing clinical progress in mental health services [38]. **Loneliness** was measured using the three-item University of California, Los Angeles (UCLA) Loneliness Scale [39]. It asks how often the respondent feels left out, isolated from others, or lacks companionship. Each question was scored on a three-point scale of ‘never or hardly ever’ [1] ‘some of the time’ [2] and ‘often’ [3]. A score of six or more was classed as lonely. This version of the UCLA is used frequently in research with AYA and is the recommended loneliness item for research and evaluation studies by the Office of National Statistics [40].

#### Social functioning

AYA in the study also completed validated measures that allowed us to explore their perceived closeness to friends, their confidence at social interaction, and embarrassment at having IBD. **The Friendship Respondent Affection Scale (FRAS)** [41] provides information on closeness the AYA feels towards their friends. Participants rated agreement with each of the items on a 9-point scale (− 4 to + 4), which has four points labelled − 3 = very much disagree, − 1 = somewhat disagree, 1 = somewhat agree, and 3 = very much agree. Sample items include “I am satisfied with my friendship with [friend’s name],” “I think my friendship with [friend’s name] is strong,” and “I hope [friend’s name] and I will stay friends”. **The Scale of Perceived Social Self-Efficacy (PSSE)** [42] is a measure of an individual’s degree of perceived social self-efficacy, defined as an individual’s degree of self- confidence involving social behaviour. The PSSE has been shown to be reliable and valid [42]. The instrument consists of 25 rationally derived items that measure the level of confidence in a variety of social situations. Responses are obtained using a five-point scale ranging from 1 (no confidence at all) to 5 (complete confidence). Examples items include “Find someone to go to lunch with” and “Put yourself in a new and different social situation”. Item scores are summed and then divided by 25, yielding total scores ranging from 1 to 5. Together, the PSSE and FRAS enabled us to examine whether AYA with IBD are [1] satisfied with their social relationships and [2] confident in their ability to make and keep effective relationships. We used the **IMPACT III (UK) Embarrassment subscale** to explore whether AYA with IBD in the current sample reported being embarrassed by their condition and how they engaged with others in social relationships. The subscale has six items and AYA in the study used a five-point scale to rate each item 1 (not at all) to 5 (very much). Example items include “Are you embarrassed because of your bowel condition?” and “Do you try and hide your IBD?”.

### Analysis plan

Summary statistics were undertaken using SPSS version 23 (SPSS Inc., Chicago, IL, USA). Three multiple regressions were also conducted, with loneliness, depressive symptoms, and anxiety as the dependent variables respectively. Based on previous work it was decided to include clinical variables that were comparable across IBD groups. Therefore, in Step 1, the block of demographic variables was entered (age, gender, and disease onset). In Step 2, we entered the clinical data linked to current disease activity and pain level. Based on the hypotheses that embarrassment of the condition, social self-efficacy, and friendship quality would be important predictors of internalising problems, those were entered in Step 3. Given the problems with over-fitting and model selection [43], bootstrapping was used to check the robustness of the findings. Bootstrapping is a method of re-sampling many times from the observed data; it allowed us to check the statistical methods by applying them to a large number of samples. For each multiple regression, the step-wise procedure was run on 1000 bootstrap replications of samples [44]. If a statistically significant coefficient is due to a genuine effect, one would expect it to be statistically significant in a high proportion of the 1000 bootstrap replicated samples, providing additional information about the generalisability of the results and reducing the likelihood of making spurious conclusions based on models that may not be stable (e.g., due to small samples or Type 1 errors from multiple testing). Because it could be argued that conducting three separate regression analyses may result in Type 1 errors, in addition to the multiple regressions, the relationships between the variables were explored using Structural Equation Modelling (SEM).

We tested two models in SEM. First, we tested the less restrictive (Full) model where all pathways were allowed to be freely estimated. Then, nonsignificant paths were fixed to zero, so the effects of nonsignificant paths could be explored through the comparison of path models. Those models were compared using the chi-square difference test. In the models, we modelled scores of depression, anxiety, and loneliness as a latent variable (‘internalising problems’). Clinical data (activity of disease and abdominal pain) were defined as categorical. In the models, associations between the predictor variables (clinical data) and the latent variable ‘internalising problems’ were modelled, exploring mediator effects of embarrassment of the condition, social self-efficacy, and friendship closeness (total scores on each measure). The models allowed for unconstrained correlations between the clinical variables.

All models were tested using MLR in Mplus 7.4. MLR was used as the estimator because it uses maximum likelihood (ML) estimation with robust chi-squares and standard errors [45] and accounts for the categorical nature of the clinical data. MLR produces the same parameter estimates as ML, but the chi-square for the model test and the standard errors for the parameters are calculated differently. MLR also provides the full maximum likelihood for missing data. MLR is robust against moderate violations of assumptions, including unmodeled heterogeneity [45].

The adequacy of the SEM models was assessed using Goodness-of-Fit (GOF) statistics. The degree of model fit was used to make interpretations about the relations between the variables and the possible mediators. GOF statistics used are the chi-square goodness of fit statistic, the comparative fit index (CFI), normed fit index (NFI), and the root mean square error of approximation (RMSEA) [46]. There are rules of thumb about acceptable levels of GOF [47], such that RMSEA should be less than 0.10 to be viewed as having a reasonable fit to the data; the CFI and NFI should exceed 0.90; the Chi-square index should be as small as possible. Standardised regression coefficients (*B*) are reported. The alpha level is set to 0.05 throughout for the SEM analyses.

## Results

Table 1 summarises the means of the study variables. Using standard cut-off points for GAD-7, PHQ-8, and UCLA, the majority of the young people in the current sample were not anxious or experienced only mild anxiety (76%), not depressed or suffered mild depression consistent with everyday stress exposure (90%), and not lonely (89%). 10% of the AYA in the study reported severe anxiety, 10% moderate/severe depression, and 11% were very often lonely (see Table 2 for details). It was the case that the same AYA tended to report all three mental health problems: those who reported higher levels of depressive symptoms were also more likely than chance to report elevated levels of anxiety and loneliness, and those with higher anxiety were also more likely than chance to be lonely, suggesting high levels of co-morbidity.

**Table 2:** Participant numbers in cut-off groups for internalising problems (depressive symptoms, anxiety, and loneliness)

	Number of AYA in sample	% Of sample
GAD-7		
Not anxious	64	49.2
Mild anxiety	35	26.9
Moderate anxiety	18	13.8
Severe anxiety	13	10.0
PHQ-8		
Not depressed	60	46.2
Other depression	57	43.8
Moderate/severe depression	13	10
Loneliness		
Lonely	14	10.8
Not lonely	116	89.2

### Associations between clinical data, feelings about social situations, and internal/mental health problems

To examine whether IBD symptoms and feelings about social relationships were related to depressive symptoms, anxiety, and loneliness, a series of multiple regressions were conducted. Table 3 summarises the results from each regression, where bootstrapping methods (1000 bootstrap replications) were used. The models were significant for anxiety (*F* = 9.88, *p* < 0.001), depressive symptoms (*F* = 9.44, *p* < 0.001), and loneliness (*F* = 2.52, *p* < 0.001). Results suggested that current high pain levels, embarrassment of the condition, and lower social self-efficacy were associated with higher self-reported anxiety and depressive symptoms, with age also associated with higher depressive symptoms. IBD symptoms were not associated with reports of loneliness, but embarrassment of the condition and lower social self-efficacy was. The bootstrapping results confirmed the robustness of the findings.

**Table 3:** Multiple regression with as predictors of depressive symptoms, anxiety, and loneliness

	Internalising problems					
	Anxiety		Depressive symptoms		Loneliness	
	Δ*R*^2^	*Β*	Δ*R*^2^	*β*	Δ*R*^2^	*β*
Step 1	.074		.094*		.023	
Gender		.005		−.065		−.039
Age		.160		.211*		.125
Years with IBD		.281		−.109		−.109
Step 2	.118*		.109***		.028	
Current disease activity		−.055		−.056		−.001
Pain Level		.281*		.279*		.089
Step 3	.273*		.405***		.130	
Embarrassment of disorder		.468**		.374**		.280*
Social self−efficacy		−.267*		−.326**		−.220*
Friendship quality				−.151		−.060
Total *R*^2^	.418*		.453**		.426	

*p < .05, **p < .001

#### SEM

The full model where all paths were freely estimated was compared against a constrained model in which the nonsignificant paths were held to zero. The full model fit the data very well (χ^2^ (df = 12) = 12.95, *p* = 0.373; CFI = 0.99; NFI = 0.97; RMSEA = 0.025 (90 confidence intervals = 0.0–0.095). The constrained model was also very good (χ^2^ (df = 21) = 20.92, *p* = 0.464; CFI = 1; NFI = 0.94; RMSEA = 0.001 (90 confidence intervals = 0.0–0.074). The chi-square difference test, Δχ2 (df = 9), 7.81, *p* > 0.05 was nonsignificant, so our interpretations focus on the full model. The inclusion of freely estimated nonsignificant paths did not change statistical significance, direction, or magnitude of effects, except for one path—that from disease activity to closeness to friends—where the path coefficient changed from − 0.24 to − 0.35, and moved from *p* < 0.01 to p < 0.001.

**Fig. 1:**
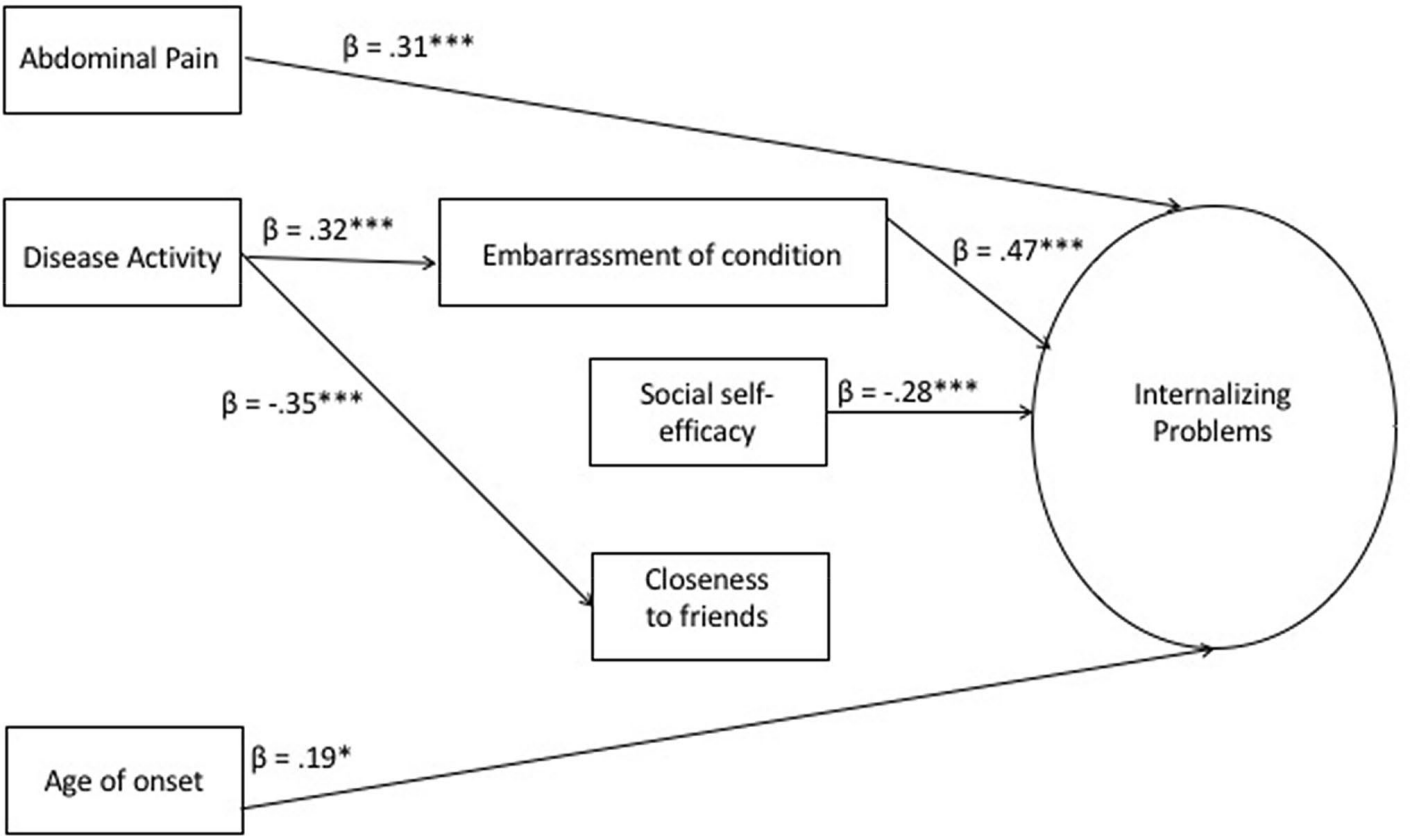
Final SEM model. *p < .01, **p < .001. The latent variable ‘Internalising problems’ is made up of total scores for loneliness (*β* = .64***), depressive symptoms (*β* = .94***), and anxiety (*β* = .89***). The path estimates included here are from the full model that allowed the nonsignificant paths to be freely estimated. Their inclusion does not have a substantive impact on the statistical significance, direction, or magnitude of effects compared to the restricted model, where the nonsignificant paths were fixed to zero. The only change was in the magnitude and significance of the path from disease activity to closeness to friends, where the path coefficient changed from − .24 to − .35 and moved from *p* < .01 to* p* < .001. Age and gender were not related to any other variables in the earlier multiple regression and so were not included in SEM analysis

Betas for significant paths from the full model are shown in Fig. 1. They show that current IBD disease activity influenced how AYA felt about their friendships and how embarrassed they were about their condition; embarrassment then influenced reports of internalising problems. Abdominal pain, disease onset, and social self-efficacy directly predicted internalising problems: the more pain, the older they were when diagnosed with IBD, and the lower their social self-efficacy, the higher their internalising problems.

## Discussion

In the current study, we investigated how IBD influenced mental health and well-being among adolescents and young adults (AYA). Consistent with previous research, IBD disease activity was found to influence mental health reporting; extending that work, we also found that it did so indirectly by affecting how young people felt about their friendships and how embarrassed they were about their condition. Essentially, having more symptoms meant AYA were more embarrassed and felt their condition affected their relationships with others. Given the unpredictable nature of the condition, patients reported being embarrassed about their symptoms and felt socially restricted because of them. Further, abdominal pain, later disease onset, and lower social self-efficacy were found to directly predict poor mental health. Those findings provide empirical evidence that how AYA feel about their social life and how that is affected by the condition contributes to their mental health and well-being. Age at onset of the disease was also important, suggesting that certain age-related experiences may be risk factors for mental health among AYA with IBD.

Consistent with previous research, there was a significant relationship between mental ill-health and elevated rates of abdominal pain. Such findings suggest that the management of pain could reduce mental ill-health for those with IBD. However, there may be mechanisms at work that were not examined in the current study: given that pain is associated with chronic fatigue in many diseases among AYA [48, 49], and fatigue is associated with depressive symptoms in patients [50, 51], the management of pain, because it is associated with fatigue, could reduce mental ill-health for those with IBD. The examination of the role of fatigue in the prospective relationship between pain and mental ill-health in AYA with IBD should be a focus of future work.

Onset of IBD was also associated with mental health: later onset was associated with poorer mental health. In another recent study [52] patients between 18 and 40 years of age at the onset of Crohn’s disease or colitis were more at risk of developing anxiety and depressive symptoms compared to patients over 40 years. Our results add to those findings, but show that among young people ages 14–25 years, it is those with later onset of the disease who report poorer mental health. Together, findings suggest there may be a sensitive period in development where onset of IBD is most problematic, and that may not start in adolescence but in young adulthood. Thus, there is a need to explore the impact of age of onset of IBD more systematically in future studies to understand whether there are specific age groups who are more at risk and why. The mechanism linking age of onset to mental health should also be explored in future prospective studies: what is it about the experience of IBD symptoms at certain ages that make mental health challenges more likely?

Consistent with our expectations, we found that disease activity was associated with mental health, but it did so indirectly via embarrassment, affecting how AYA felt about their friendships. Given the social stigma that surrounds IBD symptoms [11, 27], it is unsurprising that feelings of embarrassment and worries about friendships are elevated during times of heightened disease activity. The unpredictable nature of symptoms and the frustrating treatment and management regimes make for embarrassing social experiences and restricted social relationships. Our findings, then, suggest that addressing the social stigma that surrounds IBD and supporting AYA through real and perceived relationships difficulties are important considerations for intervention designs.

### Implications of the findings

The findings suggest a clear need for screening of mental health for AYA with IBD, and the use of standardised instruments with good sensitivity to monitor and improve individual outcomes over time. Understanding the role that social relationships and stigma have on the individual and at key points in the disease activity is important. One outcome of our findings could be the systematic screening of AYA with IBD, to create opportunities to consider appropriate preventive and supportive interventions for early signs of distress, rather than waiting to respond until a more severe psychological impairment emerges. Where screening is not possible, creating opportunities to discuss mental health challenges with each patient will be important, making discussion of mental health a natural part of the clinical conversation. Where regular screening is possible, there will be opportunities to monitor any changes in the mental health care needs of AYA patients over time, and for research to explore the impact of new disease-modifying therapies on mental health outcomes. Given the finding that embarrassment mediates the relationship between IBD activity and mental health outcome and impacts perceived closeness to friends, we recommend that these be seen as potential foci in therapeutic work for youth with IBD and also in future research.

## Limitations of the current study and future work

Given the increased risk of mental health problems among AYA with IBD, it is possible that those individuals in the sample who had an early diagnosis of IBD had also undergone treatment for mental health problems, particularly in centres where there is a holistic approach to health care. Thus, findings from the current study that later diagnosis among AYA is associated with poorer mental health may reflect the fact that treatment of mental health problems is already happening for those with earlier diagnosis. That said, we are not aware of any special mental health support available in those clinics we recruited from, suggesting that the mental health support came from elsewhere, most likely from their parents and friends. Future research will need to explore that possibility and look at individual context more explicitly.

## Conclusion

In the current study, depressive symptoms, anxiety, and loneliness were common experiences for some adolescents and young adults (AYA) with IBD. The findings suggest that aspects of the condition are associated with mental health challenges in the current cohort, but that embarrassment surrounding the condition and the perceived impact on friendships were also important. We conclude by arguing that patients should have opportunities to discuss their mental health within the usual clinical dialogue, such that clinics help AYA with all challenges associated with living with IBD, including the impacts it has on social relationships and quality of life.
